# Differential expression of RNA exosome subunits in the amphibian *Lithobates catesbeianus* during reproductive and non-reproductive periods

**DOI:** 10.1186/s13104-019-4077-7

**Published:** 2019-01-21

**Authors:** J. S. Luz, B. H. Caneguim, A. Baggio, M. M. Santoni, C. C. Helbing, S. R. Valentini, E. Sasso-Cerri, C. C. Oliveira

**Affiliations:** 1 School of Pharmaceutical Sciences, São Paulo State University (UNESP), Araraquara, Brazil; 2 Department of Morphology and Genetics, Federal University of São Paulo (UNIFESP), São Paulo, Brazil; 3 Present Address: Federal University of Triângulo Mineiro (UFTM)-Iturama University Campus (UFTM-CIT), Iturama, Brazil; 4 Department of Biochemistry and Microbiology, University of Victoria, Victoria, BC Canada; 5 Department of Morphology, Dental School of São Paulo State University, Araraquara, Brazil; 6 Department of Biochemistry, Chemistry Institute, University of São Paulo, São Paulo, Brazil

**Keywords:** RNA exosome, Protein localization, *Lithobates catesbeianus*, Tissue expression

## Abstract

**Objective:**

The RNA exosome is an evolutionarily conserved 3′–5′ exoribonucleolytic protein complex involved in processing and degradation of different classes of nuclear and cytoplasmic RNAs, and, therefore, important for the posttranscriptional control of gene expression. Despite the extensive in vivo functional studies and the structural data on the RNA exosome, few studies have been performed on the localization and expression of exosome subunits during gametogenesis, process during which gene expression is largely controlled at the posttranscriptional level.

**Results:**

We report the identification of exosome subunits in *Lithobates catesbeianus* and analysis of the differential subcellular localization of RNA exosome core and catalytic subunits in testis cells. In addition, we show seasonal differences in the expression levels of four exosome subunits in different organs. In addition to being part of the RNA exosome complex, its subunits might participate independently of the complex in the control of gene expression during seasonal variation in bullfrog tissues. These results may be relevant for other eukaryotic species.

**Electronic supplementary material:**

The online version of this article (10.1186/s13104-019-4077-7) contains supplementary material, which is available to authorized users.

## Introduction

The eukaryotic RNA exosome is an essential multisubunit ribonuclease complex that participates in degradation or processing of all classes of RNA in the nucleus and cytoplasm. Nuclear RNA exosome is involved in 3′–5′ processing of rRNAs, snRNAs and snoRNAs, degradation of hypomodified tRNAs, and cryptic unstable transcripts (CUTs), whereas cytoplasmic RNA exosome is responsible for the degradation of both normal and aberrant mRNA species, in addition to being involved in degradation of interfering RNAs and ncRNAs [1, 2].

The RNA exosome was first identified in yeast and subsequently found in other eukaryotes as well as in archaea [3–6]. The eukaryotic RNA exosome is composed of eleven different subunits, nine of which compose the exosome core (Exo9). Six of the core subunits form the RNase PH ring (RRP41, RRP42, RRP43, RRP45, RRP46 and MTR3) and three S1/KH RNA binding proteins (RRP4, RRP40 and CSL4) form the cap, which binds to one side of the PH ring. The eukaryotic RNA exosome catalytic activity, however, is dependent on the presence of the additional subunits RRP44/DIS3 and RRP6, which form Exo10 and Exo11, respectively [7]. RRP44 is a processive hydrolytic RNase R homologue, present both in the yeast nuclear and cytoplasmic exosome (Exo10). The eleventh subunit RRP6 is a distributive hydrolytic RNase homologue of RNase D, associated only with the nuclear exosome in yeast (Exo11) [8].

*Saccharomyces cerevisiae* exosome subunit yRRP6 has been shown to have its expression levels decreased from vegetative growth to meiosis and spore formation [9]. Based on the RNA exosome participation in the maturation and degradation of different RNAs in the cells, studies on the localization and function of the exosome in vertebrates could shed some light on its activity during gametogenesis, a finely regulated process that requires a precise and highly ordered control of gene expression to maintain the cell cycle progression and morphogenetic changes, and to produce functional gametes.

As in other amphibians, the spermatogenesis of the bullfrog *Lithobates catesbeianus* is characterized by morphofunctional testicular changes that occur in the seasonal reproductive cycle, divided in two phases: quiescent (winter) and active (summer), making the bullfrog testis an interesting model to study spermatogenesis [10–12].

In this work, we used *L. catesbeianus* for the study of the RNA exosome in testis during spermatogenesis and in non-reproductive tissues. We analyzed the localization of RNA exosome subunits in testis, focusing on interstitial, and the primordial germ cells (PGCs). Our data show that, except for RRP6, the localization of exosome subunits is similar in the testicular cells during reproductive and quiescent periods in PGCs. In addition, contrary to testis, a significant variation in the levels of exosome subunits was detected when analyzing non-reproductive tissues in the same periods, suggesting that individual RNA exosome subunits might play different roles during cell differentiation.

## Main text

### Materials and methods

#### Animals

Ten adult male bullfrogs (*Lithobates catesbeianus*) were obtained from RANAMAT frog farm in Matão (São Paulo, Brazil), in March (summer) and July (winter). The animals were divided into summer (SG; n = 5) and winter (WG; n = 5) groups, respectively.

#### Sample preparation and histological procedures

The animals were anaesthetized with 10% chloral hydrate for the removal of the different organs, which were stored at − 80 °C for protein extraction and immunoblot analysis. The remaining contralateral testes were fixed in 4% formaldehyde/0.1 M sodium phosphate pH 7.4. Testes were dehydrated in alcohol and embedded in paraffin or historesin. The historesin sections were stained by Gill’s hematoxylin and eosin—H.E. [13], and the paraffin sections were subjected to immunohistochemical reactions as described [11].

The primary polyclonal antibodies against human antigens used (Additional file 1: Table S1) had been validated by Human Protein Atlas portal [14]. The histological analyses were performed using a camera (DP-71; Olympus) attached to a light microscope (BX-51; Olympus).

#### Protein extraction and immunoblot analysis

Bullfrog frozen organs were homogenized directly in lysis buffer (50 mM Tris pH 8.0, 300 mM NaCl, 1 mM EDTA, 1 mM DTT, 10% glycerol, 1 mM MgCl_2_, 1 mM phenylmethylsulfonyl fluoride (PMSF), and 5 ng/mL of the protease inhibitors Pepstatin, Leupeptin, Aprotinin, Antipain and Chymostatin). Crude extracts were clarified by centrifugation at 13,000 rpm for 20 min. Protein concentrations were determined with bicinchoninic acid (Sigma-Aldrich).

20 μg of total protein extracts were subjected to SDS–PAGE and transference to nitrocellulose membranes (GE healthcare). Western blot was performed by incubation of the membranes with the primary antibodies against human proteins and anti-rabbit secondary antibody. Anti-human actin was used as internal control. The immunoblots were developed using the Luminata Forte Western HRP Substrate (Millipore) and quantification was performed using ImageJ software.

#### Sequence alignment

CLUSTAL Omega [15] was used for the alignment of sequences from *L. catesbeianus*, *Xenopus* and human.

### Results

**Fig. 1:**
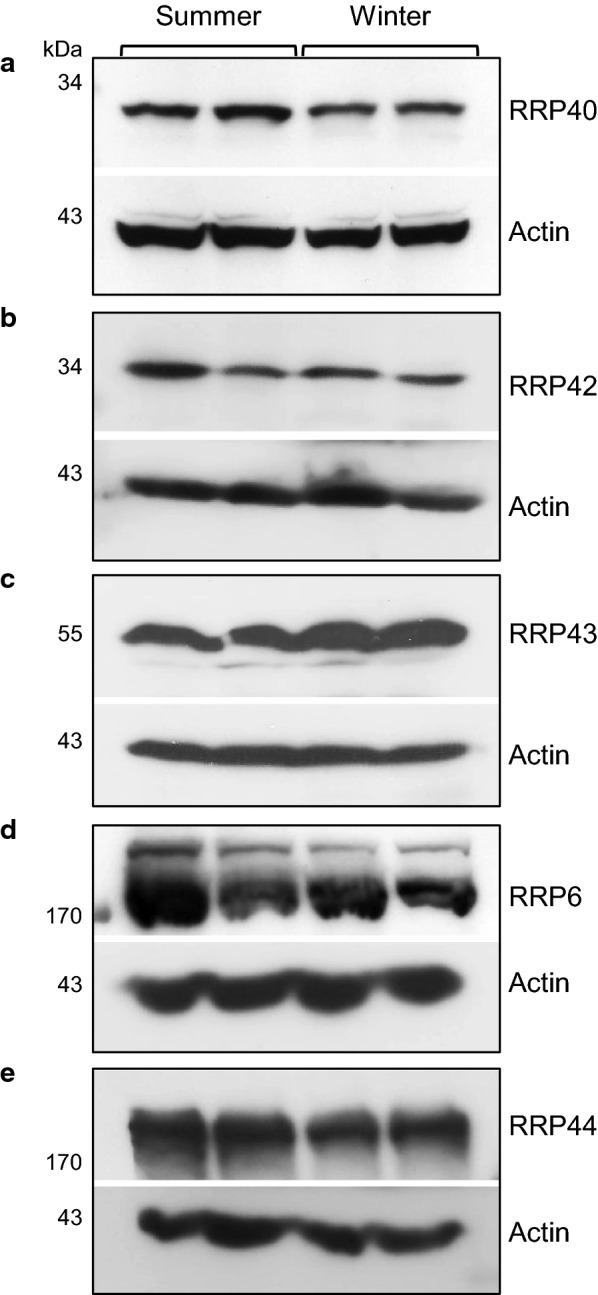
Expression of *L. catesbeianus* RNA exosome subunits in testis in reproductive (summer) and quiescent (winter) periods. The total testis extracts were obtained from two biological samples in both periods, and subjected to western blot for detection of the RNA exosome subunits RRP40 (**a**), RRP42 (**b**), RRP43 (**c**), RRP6 (**d**) and RRP44 (**e**). *β*-*actin* was used as an internal control of the total protein loaded on gels

To study the RNA exosome during spermatogenesis in *L. catesbeianus* testis, we first compared the amino acid sequences of the bullfrog RNA exosome subunits, obtained from a Bullfrog Annotation Resource for the Transcriptome (BART) derived from de novo assembled bullfrog RNA-seq data [16], to those of *Xenopus* and human (Additional file 1: Figures S1–S4). The bullfrog exosome subunits RRP40, CSL4, RRP41, RRP42, RRP43, RRP44, and RRP6, which had been identified in the BART data, retain at least 69% identity and 83% similarity with *Xenopus tropicalis* or *Xenopus laevis* homologs, and 64% identity and 80% similarity with *Homo sapiens* (Additional file 1: Table S2). This high sequence conservation indicated that antibodies against human exosome subunits could be used to identify *L. catesbeianus* homologs. Confirming the evolutionary conservation of these proteins, antibodies against RRP40, RRP42, RRP43, RRP6 and RRP44 detected single proteins in the *L. catesbeianus* extracts (Fig. 1).

To determine whether there are changes in the exosome subunits expression in testis during the reproductive and quiescent periods, two independent biological samples from Summer or Winter, respectively, were analyzed by western blot. These five exosome subunits were expressed in both periods with no strong difference in their levels (Fig. 1), but interestingly, their expression levels are very high in testis, similar to those of actin, confirming the important role of the exosome in the control of gene expression.

The amino acid sequences indicated that LcRRP40 and LcRRP42 have 27 and 32 kDa, respectively, in agreement with the bands visualized on the western blot (Fig. 1). LcRRP43 is predicted to have 30 kDa, but it appears as a 55 kDa protein (Fig. 1). LcRRP6 and LcRRP44 should have 96 kDa and 109 kDa, respectively, but are visualized as much larger proteins (Fig. 1). These results suggest that these proteins undergo posttranslational modification. This hypothesis was strengthened by the observation that recombinant LcRRP43 runs as the expected 30 kDa protein (data not shown). Importantly, putative phosphorylation, sumoylation and ubiquitination sites were identified in these sequences (Additional file 1: Figures S1–S4).

Testes of bullfrog of the groups of animals used in this work showed the characteristic testicular morphofunctional changes in the non-reproductive and reproductive periods, winter and summer, respectively (Additional file 1: Figure S5). In the bullfrog testes only of the summer group, the developed interstitial tissue showed numerous Leydig-like interstitial cells (Additional file 1: Figure S5A), characteristic of an active steroidogenic process in testis.

**Fig. 2:**
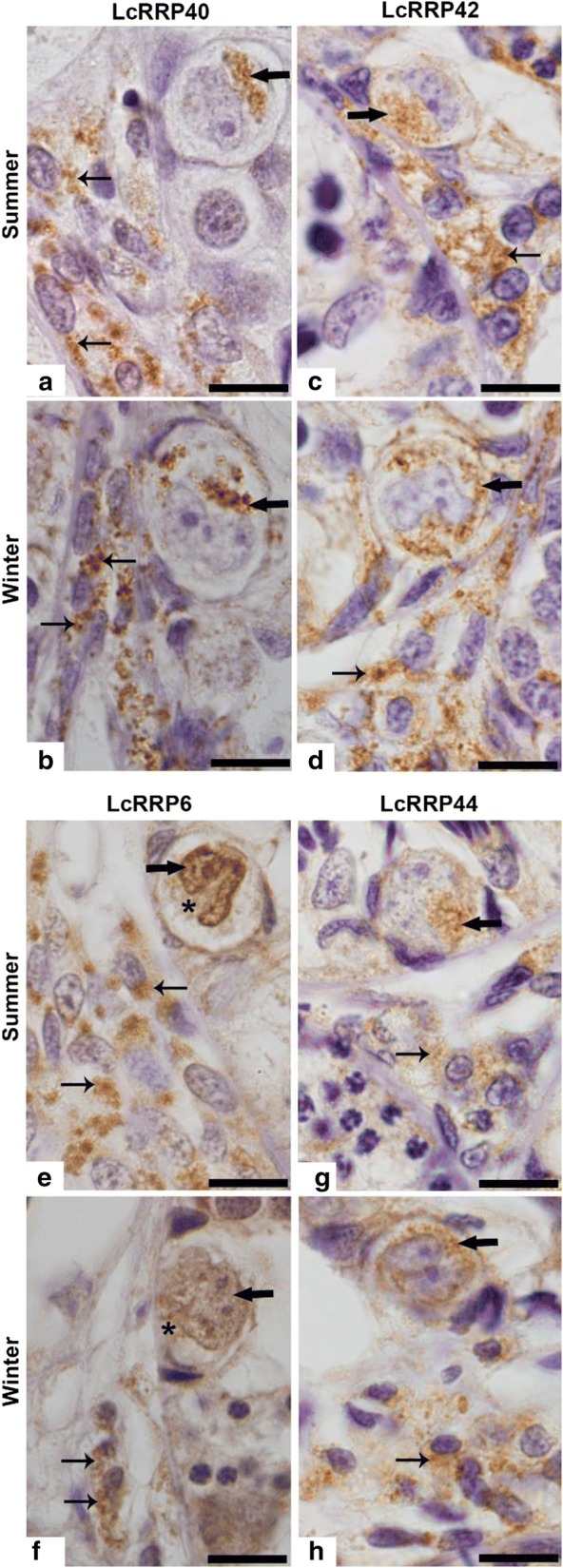
Photomicrographs of seminiferous lobules of *L. catesbeianus* from summer groups (**a**, **c**, **e**, **g**) and winter groups (**b**, **d**, **f**, **h**,) submitted to immunohistochemistry for detection of LcRRP40 (**a**, **b**), LcRRP42 (**c**, **d**), LcRRP6 (**e**, **f**), and LcRRP44 (**g**, **h**). Note cytoplasmic immunolabeling (arrows) in the PGCs and in the interstitial cells (thin arrows) for: LcRRP40, LcRRP42, and LcRRP44 in both groups. A strong LcRRP6 immunolabeling is observed in the nucleus of PGCs (arrows) of Summer in comparison to Winter, and a weak or absent immunolabeling is observed in the cytoplasm of these cells (asterisks). LcRRP6 immunolabeling (thin arrows) is observed in the cytoplasm of interstitial cells in both periods. Bars: 10 µm

To study the RNA exosome during spermatogenesis, we analyzed the subcellular localization of its subunits LcRRP40, LcRRP42, LcRRP6 and LcRRP44 in testes of *L. catesbeianus* during the reproductive and quiescent periods, focusing on PGCs and interstitial cells because of the morphological changes they undergo in these periods [10, 11]. The results show that the exosome subunits were present in the cytoplasm of both interstitial cells and PGCs, in both periods (Fig. 2), suggesting that in these cells the exosome complex containing Rrp44 (Exo10) is mainly concentrated in the cytoplasm. Despite a weak immunolabeling in the cytoplasm of PGCs, LcRRP6 localized mainly to the nucleus, including nucleolus (Fig. 2). Interestingly, similar subcellular localization has been described for RRP6 in human, fly and trypanosome cells [17–19].

**Fig. 3:**
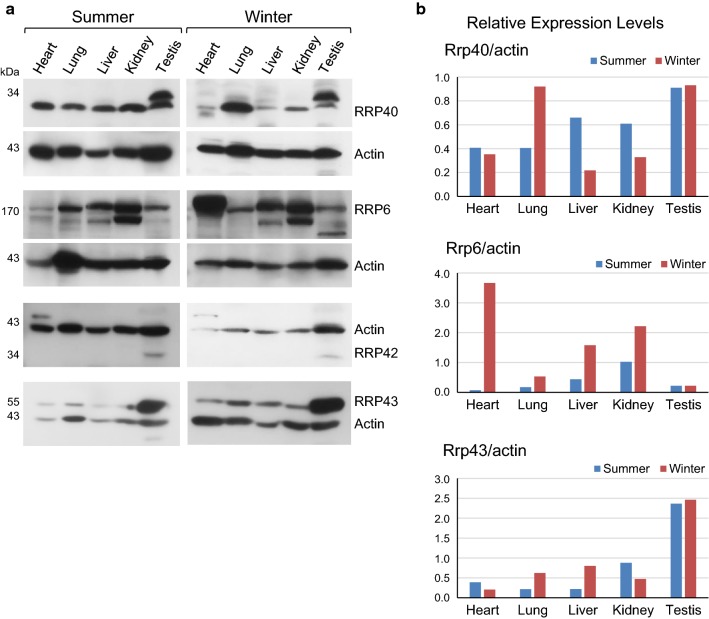
Expression of *L. catesbeianus* RNA exosome subunits in heart, lung, liver, kidney and testis in summer and winter periods. **a** Total extracts were obtained from groups of animals in reproductive or quiescent periods, and western blot for the detection of the exosome subunits RRP40, RRP6, RRP42 and RRP43 was performed. **b** Relative expression levels of exosome subunits after quantification of bands, normalized to *β*-*actin*. Data are representative of two biological samples

To further analyze the RNA exosome subunits in *L. catesbeianus*, we compared the expression levels of its subunits in non-reproductive tissues, in samples from the reproductive or quiescent periods. Surprisingly, the expression levels of the exosome subunits vary significantly in non-reproductive tissues (Fig. 3). In summer, LcRRP40 expression was similar among the non-reproductive tissues and showed a double band in testis (Fig. 3). In winter, there is an expressive reduction in the levels of LcRRP40 in heart, liver and kidney, and an increased level of the expression in lung. In testis, the level of the upper band of LcRRP40 remained unchanged, whereas the lower band showed a strong decrease. The upper band corresponds to the band visualized in Fig. 1 (in that gel, the lower band was only visible after long exposure). These results suggest a differential expression of LcRRP40 in response to seasonal conditions. In addition, the two bands suggest that LcRRP40 may undergo posttranslational modification in testis, indicating that subunits modifications might play a role in controlling the function of the exosome in different organs.

The levels of LcRRP6 also showed large variation when comparing organs and seasons (Fig. 3). Strikingly, however, the changes in expression levels of LcRRP40 and LcRRP6 are not correlated. In heart, LcRRP40 levels are high in summer but lower drastically in winter, the opposite of LcRRP6. Interestingly, there is an increase in the LcRRP6 levels in heart, liver and kidney in winter, whereas in lung and testis its levels remain unchanged (Fig. 3). In addition, a major band of LcRRP6 is detected in all organs in summer and winter groups, but in liver and kidney, a second band is also detected, which might correspond to an isoform of this protein.

LcRRP42 was only detected in testis, showing no seasonal expression variation (Fig. 3). However, a higher band was detected in heart, which may also be due to a tissue-specific isoform. Similar to LcRRP42, LcRRP44 was not detected in non-reproductive organs (data not shown).

LcRRP43 was expressed in all organs in both periods, but in much higher levels in testis than in the non-reproductive organs (Fig. 3). Although the levels of LcRRP43 did not change seasonally in testis, its levels increased in winter in the non-reproductive tissues (Fig. 3).

### Discussion

The constitutive expression of the RNA exosome in *L. catesbeianus* testis confirms its important role in gene expression regulation. The presence of exosome core subunits in the cytoplasm in the reproductive period, while LcRRP6 was detected in nuclei, suggests that LcRRP6 might have functions independent of the exosome. Confirming our results, hRRP6 has been suggested to have a specific role in RNA processing during the development of spermatogonia [20], and shown to localize to nucleoli of mouse spermatogonia, but become undetectable when cells enter spermiogenesis [21].

The products of alternative splicing have been identified for various RNA exosome subunits in different species [22–24], strengthening the hypothesis that this might also happen in *L. catesbeianus*. Although the structure of the eukaryotic RNA exosome indicates that all subunits are expressed at similar levels, variations in mRNA and protein levels of exosome subunits have been identified in human tissues [14, 24]. In bullfrog, the subunits of the exosome LcRRP42, LcRRP43, LcRRP40, and LcRRP6 are not expressed at similar levels in different tissues. Furthermore, the levels of some subunits undergo extreme variations through the seasons.

These intriguing results suggest that the RNA exosome composition and activity can be tissue specific, and its activity can be modulated by the integrity of the complex, as well as by the cellular compartmentalization or by posttranslational modifications of the exosome subunits. Based on the striking sequence conservation of the RNA exosome subunits, the results shown here may be relevant not only to amphibians, but also to other organisms.

## Limitations

Environmental changes resulted in lack of additional winter samples. Although it would be possible to obtain summer frogs to perform additional experiments and statistically analyze the data, the warm following winter did not cause reproductive changes in the animals, preventing us from obtaining comparable winter frogs. Consequently, the small number of samples is the major limitation of this work. Our results of RNA exosome subunits localization and differential expression are however, strengthened by published studies of other organisms.

## Additional file

**Additional file 1: Figure S1.** Multiple sequence alignment of LcRRP40. The full sequence of LcRRP40 and its putative orthologs from *Xenopus* and human were aligned. Numbers correspond to amino acid position in each protein. Proteins access numbers: Xt (*Xenopus tropicalis*—XP_002936379), Xl (*Xenopus laevis*—NP_001089320.1) and Hs (*Homo sapiens*—NP_057126.2). (*), identity; (:), strong similarity; (.), weak similarity. The amino acids residues involved in the RNA interactions, in the subunits interactions and those that are possible targets of posttranslational modifications are highlighted. CLUSTAL Omega was used for the sequence alignment [15]. **Figure S2.** Multiple sequence alignment of LcRRP6. The full sequence of LcRRP6 and its putative orthologues from *Xenopus* and human were aligned. Numbers correspond to amino acid position in each protein. Proteins access numbers: Xt (*Xenopus tropicalis*—XP_012821790), Xl (*Xenopus laevis*—NP_001084822.1) and Hs (*Homo sapiens*—NP_001001998.1). (*), identity; (:), strong similarity; (.), weak similarity. The amino acids residues present in the active site and those that are probable targets of posttranslational modifications were highlighted. CLUSTAL Omega was used for the sequence alignment [15]. **Figure S3.** Multiple sequence alignment of LcRRP42. The full sequence of LcRRP42 and its putative orthologues from *Xenopus* and human were aligned. Numbers correspond to amino acid position in each protein. Proteins access numbers: Xt (*Xenopus tropicalis*—NP_001032342), Xl (*Xenopus laevis*—NP_001086766.1) and Hs (*Homo sapiens*—BC012831). (*), identity; (:), strong similarity; (.), weak similarity. The amino acids residues involved in the interactions with the hexamer and RRP4 were highlighted. CLUSTAL Omega was used for the sequence alignment [15]. **Figure S4.** Multiple sequence alignment of LcRRP43. The full sequence of LcRRP43 and its putative orthologs from *Xenopus* and human were aligned. Numbers correspond to amino acid position in each protein. Proteins access numbers: Xt (*Xenopus tropicalis*—NP_001007919), Xl (*Xenopus laevis*—NP_001079391.1) and Hs (*Homo sapiens*—NP_852480.1). (*), identity; (:), strong similarity; (.), weak similarity. The amino acids residues involved in the interactions with the hexamer and that are probably targets of posttranslational modifications were highlighted. CLUSTAL Omega was used for the sequence alignment [15]. **Figure S5.** Photomicrographs of seminiferous lobules of *L. catesbeianus* from summer group (A, B) and winter group (C, D) stained by H&E. The seminiferous lobules (SL) from winter (C) are larger than those from summer (A). However, the interstitial tissue from summer (A) is more developed and contains numerous Leydig cells (asterisks) than that from winter (C). Both groups (B, D) show primordial germ cells (PGCs; arrows) located in the lobular periphery. In high magnification, PGCs with large polymorphous nucleus (n) and evident nucleoli (thin arrow) are surrounded by Sertoli cells (S). Bars: A, C—45 µm; B, D—25 µm; insets—6 µm. **Table S1.** Antibodies against human homologs used to identify bullfrog exosome subunits. **Table S2.** Sequence conservation of *Lithobatescates beianus* RNA exosome subunits relative to *Homo sapiens, Xenopus laevis,* or *Xenopus tropicalis*.
