# Supplementary material for: Differential expression of RNA exosome subunits in the amphibian Lithobates catesbeianus during reproductive and non-reproductive periods
Source: BMC Res Notes. 2019 Jan 21;12:46. doi: 10.1186/s13104-019-4077-7 (PMC6341637; doi:10.1186/s13104-019-4077-7)
Supplement: Supplementary file 1 — Additional file 1: Figure S1. Multiple sequence alignment of LcRRP40. The full sequence of LcRRP40 and its putative orthologs from Xenopus and human were aligned. Numbers correspond to amino acid position in each protein. Proteins access numbers: Xt (Xenopus tropicalis—XP_002936379), Xl (Xenopus laevis—NP_001089320.1) and Hs (Homo sapiens—NP_057126.2). (*), identity; (:), strong similarity; (.), weak similarity. The amino acids residues involved in the RNA interactions, in the subunits interactions and those that are possible targets of posttranslational modifications are highlighted. CLUSTAL Omega was used for the sequence alignment [15]. Figure S2. Multiple sequence alignment of LcRRP6. The full sequence of LcRRP6 and its putative orthologues from Xenopus and human were aligned. Numbers correspond to amino acid position in each protein. Proteins access numbers: Xt (Xenopus tropicalis—XP_012821790), Xl (Xenopus laevis—NP_001084822.1) and Hs (Homo sapiens—NP_001001998.1). (*), identity; (:), strong similarity; (.), weak similarity. The amino acids residues present in the active site and those that are probable targets of posttranslational modifications were highlighted. CLUSTAL Omega was used for the sequence alignment [15]. Figure S3. Multiple sequence alignment of LcRRP42. The full sequence of LcRRP42 and its putative orthologues from Xenopus and human were aligned. Numbers correspond to amino acid position in each protein. Proteins access numbers: Xt (Xenopus tropicalis—NP_001032342), Xl (Xenopus laevis—NP_001086766.1) and Hs (Homo sapiens—BC012831). (*), identity; (:), strong similarity; (.), weak similarity. The amino acids residues involved in the interactions with the hexamer and RRP4 were highlighted. CLUSTAL Omega was used for the sequence alignment [15]. Figure S4. Multiple sequence alignment of LcRRP43. The full sequence of LcRRP43 and its putative orthologs from Xenopus and human were aligned. Numbers correspond to amino acid position in each protein. Pr [file 13104_2019_4077_MOESM1_ESM.pptx]

## Slide 1
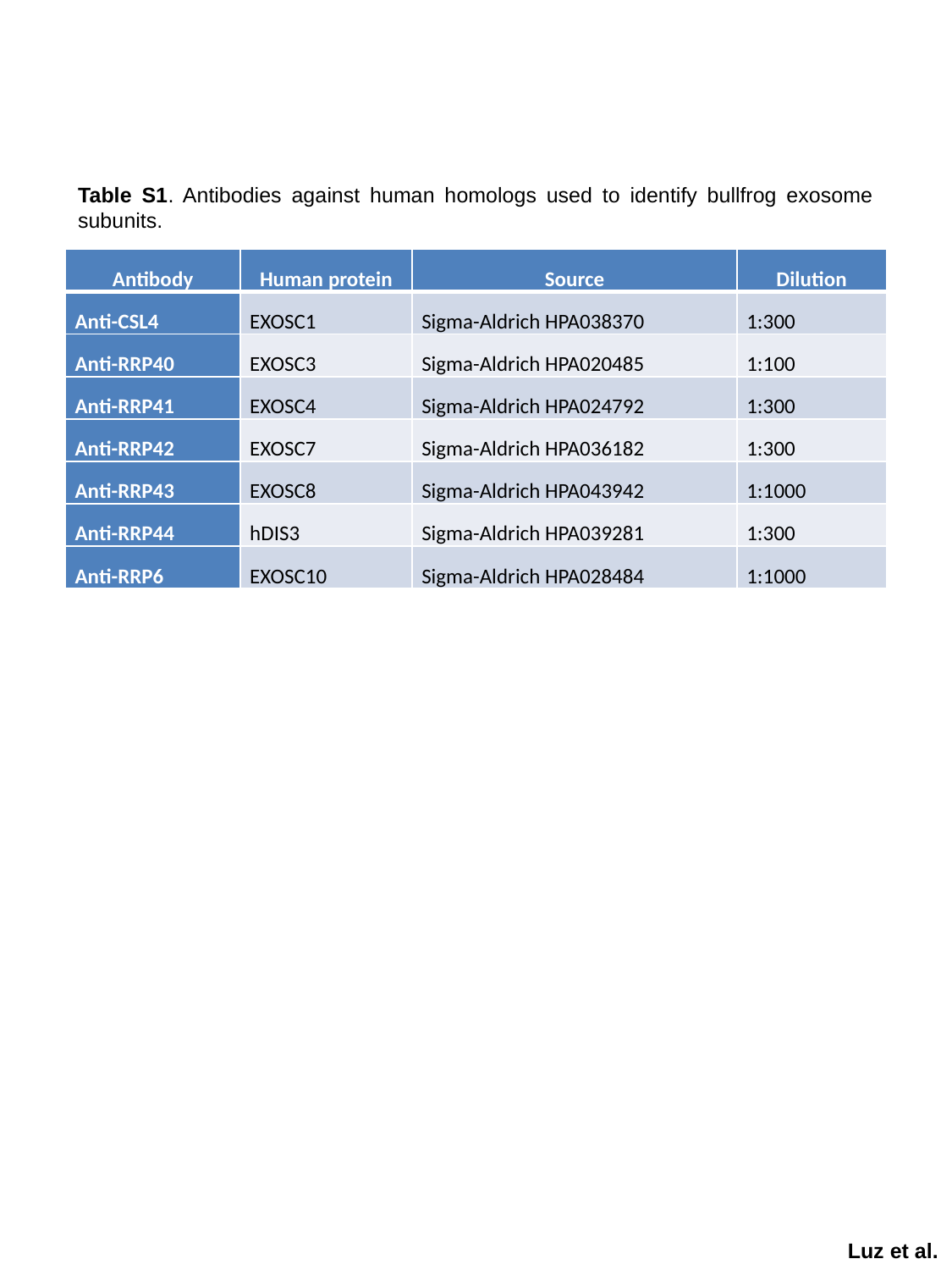

Table S1. Antibodies against human homologs used to identify bullfrog exosome subunits.
| Antibody | Human protein | Source | Dilution |
| --- | --- | --- | --- |
| Anti-CSL4 | EXOSC1 | Sigma-Aldrich HPA038370 | 1:300 |
| Anti-RRP40 | EXOSC3 | Sigma-Aldrich HPA020485 | 1:100 |
| Anti-RRP41 | EXOSC4 | Sigma-Aldrich HPA024792 | 1:300 |
| Anti-RRP42 | EXOSC7 | Sigma-Aldrich HPA036182 | 1:300 |
| Anti-RRP43 | EXOSC8 | Sigma-Aldrich HPA043942 | 1:1000 |
| Anti-RRP44 | hDIS3 | Sigma-Aldrich HPA039281 | 1:300 |
| Anti-RRP6 | EXOSC10 | Sigma-Aldrich HPA028484 | 1:1000 |
Luz et al.

## Slide 2
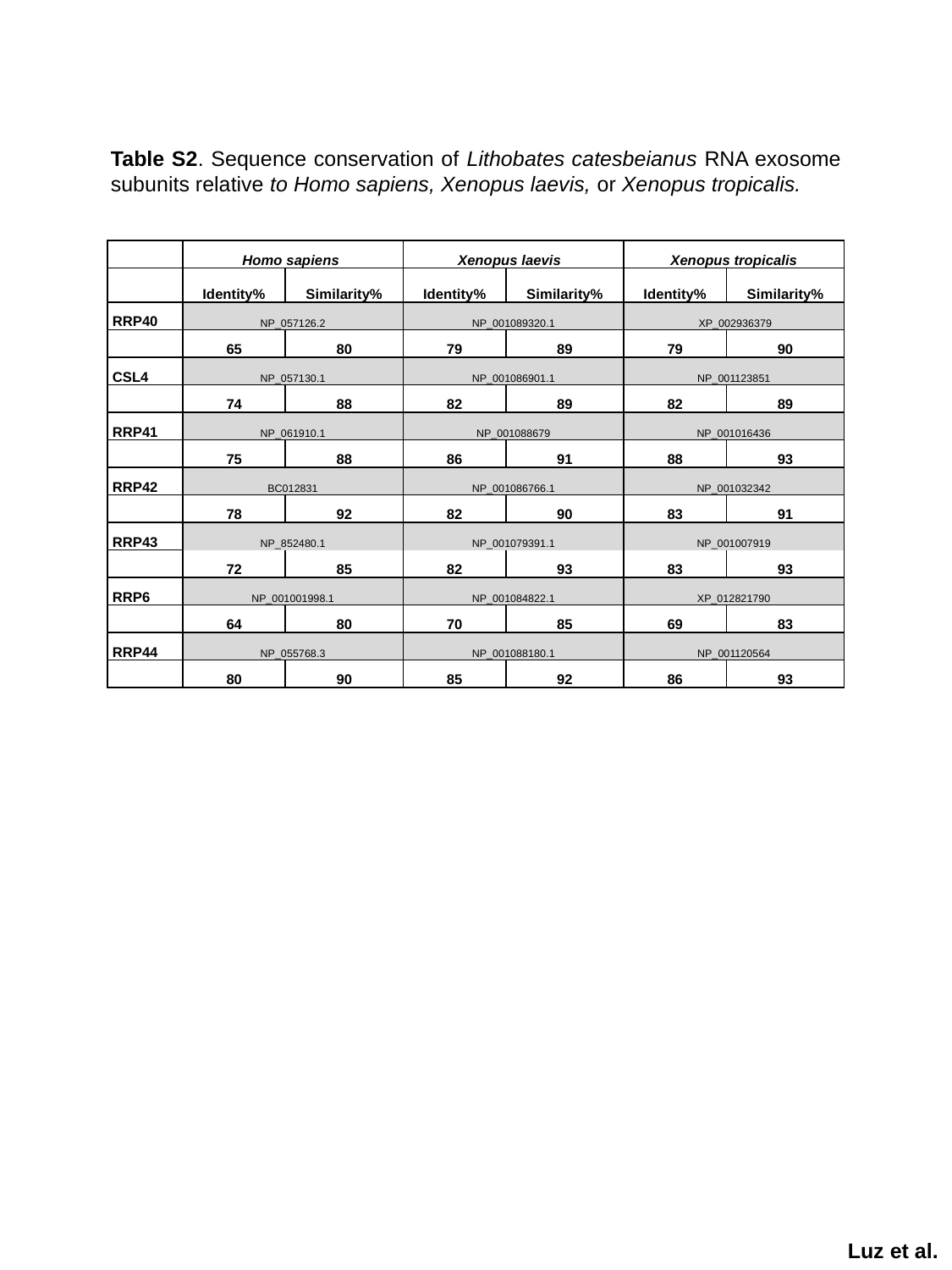

Table S2. Sequence conservation of Lithobates catesbeianus RNA exosome subunits relative to Homo sapiens, Xenopus laevis, or Xenopus tropicalis.
| | Homo sapiens | | Xenopus laevis | | Xenopus tropicalis | |
| --- | --- | --- | --- | --- | --- | --- |
| | Identity% | Similarity% | Identity% | Similarity% | Identity% | Similarity% |
| RRP40 | NP\_057126.2 | | NP\_001089320.1 | | XP\_002936379 | |
| | 65 | 80 | 79 | 89 | 79 | 90 |
| CSL4 | NP\_057130.1 | | NP\_001086901.1 | | NP\_001123851 | |
| | 74 | 88 | 82 | 89 | 82 | 89 |
| RRP41 | NP\_061910.1 | | NP\_001088679 | | NP\_001016436 | |
| | 75 | 88 | 86 | 91 | 88 | 93 |
| RRP42 | BC012831 | | NP\_001086766.1 | | NP\_001032342 | |
| | 78 | 92 | 82 | 90 | 83 | 91 |
| RRP43 | NP\_852480.1 | | NP\_001079391.1 | | NP\_001007919 | |
| | 72 | 85 | 82 | 93 | 83 | 93 |
| RRP6 | NP\_001001998.1 | | NP\_001084822.1 | | XP\_012821790 | |
| | 64 | 80 | 70 | 85 | 69 | 83 |
| RRP44 | NP\_055768.3 | | NP\_001088180.1 | | NP\_001120564 | |
| | 80 | 90 | 85 | 92 | 86 | 93 |
Luz et al.

## Slide 3
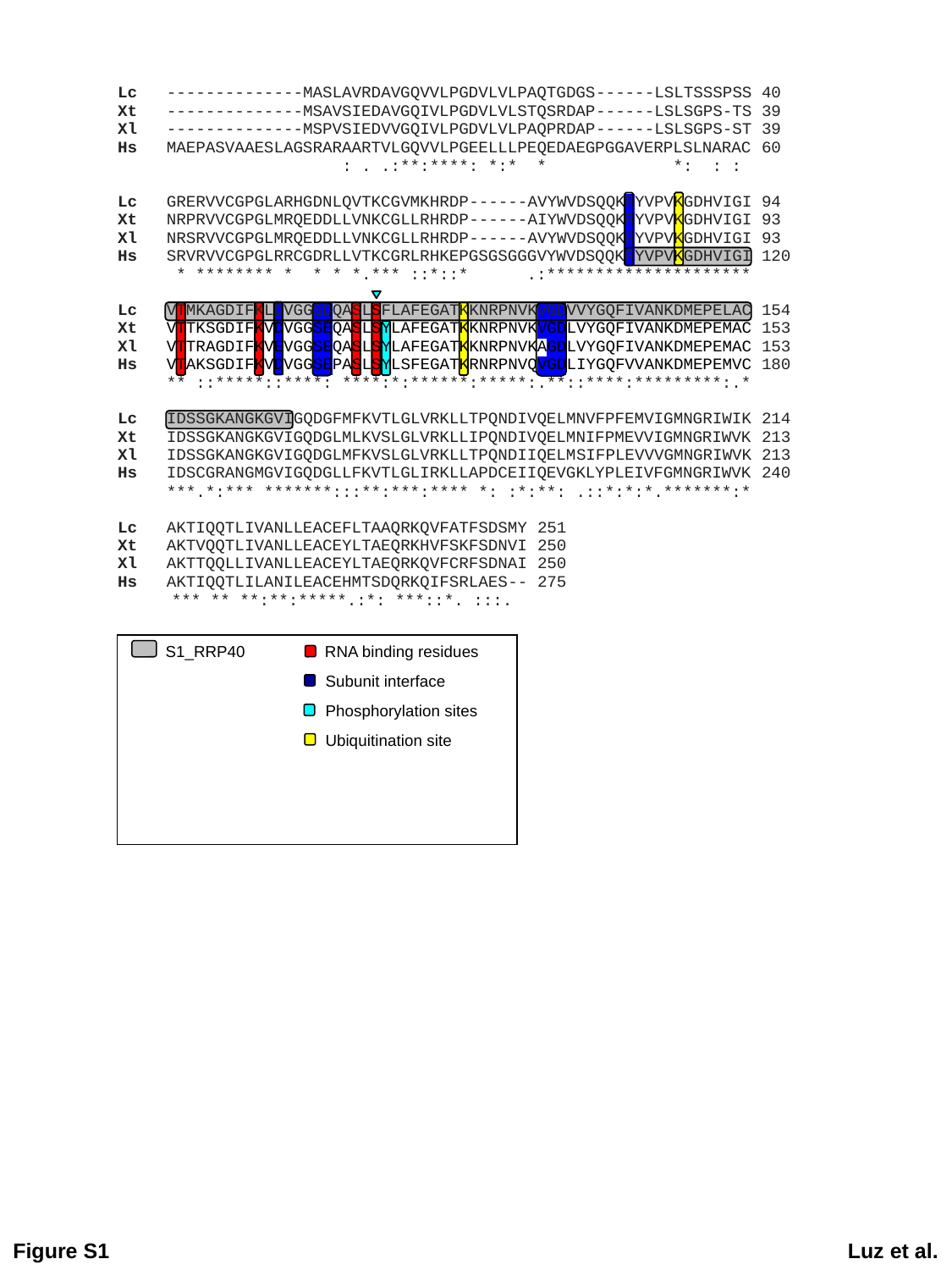

S1_RRP40 RNA binding residues
 Subunit interface
 Phosphorylation sites
 Ubiquitination site
Figure S1
Luz et al.

## Slide 4
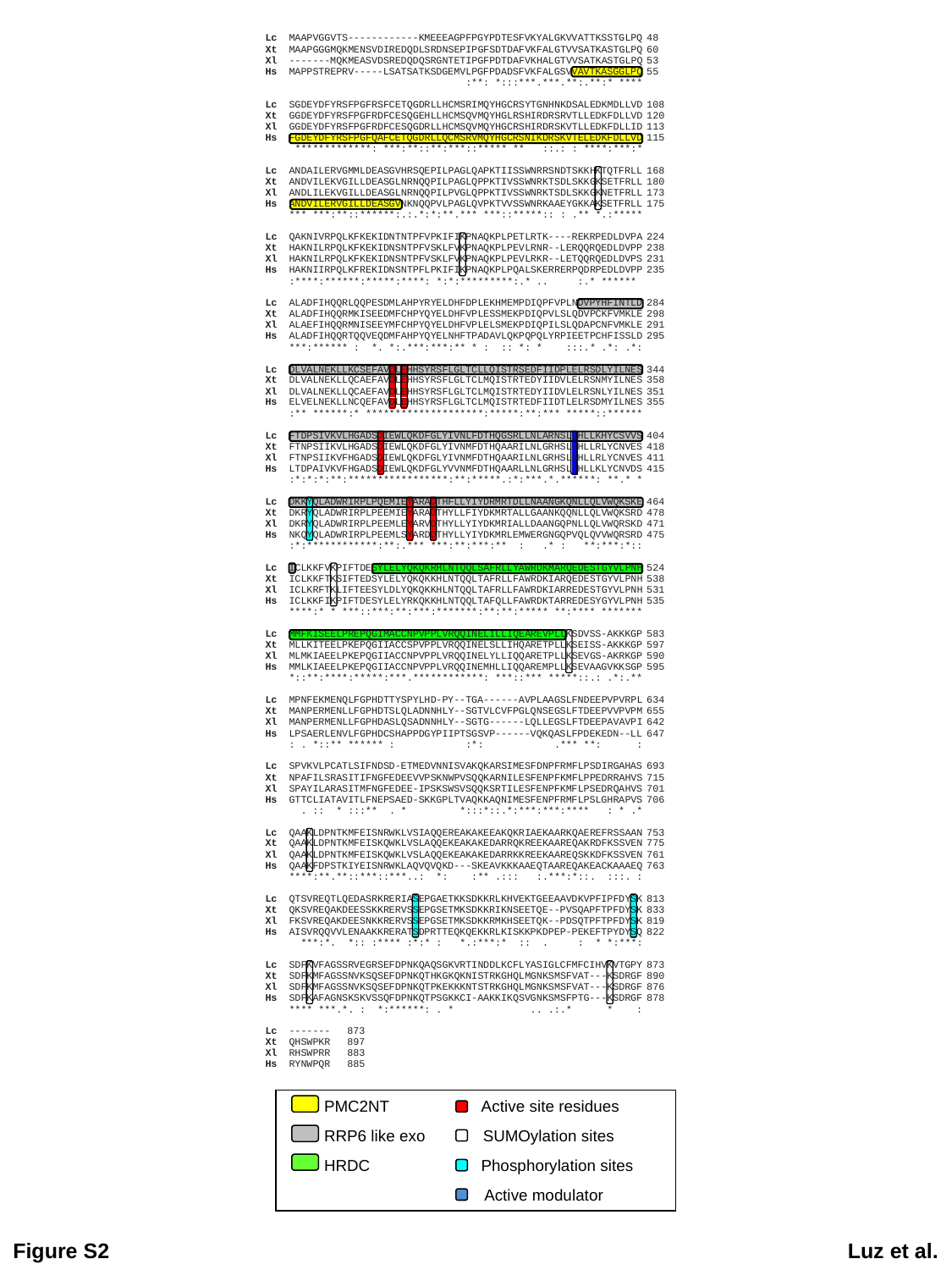

PMC2NT Active site residues
 RRP6 like exo SUMOylation sites
 HRDC Phosphorylation sites
 Active modulator
Figure S2
Luz et al.

## Slide 5
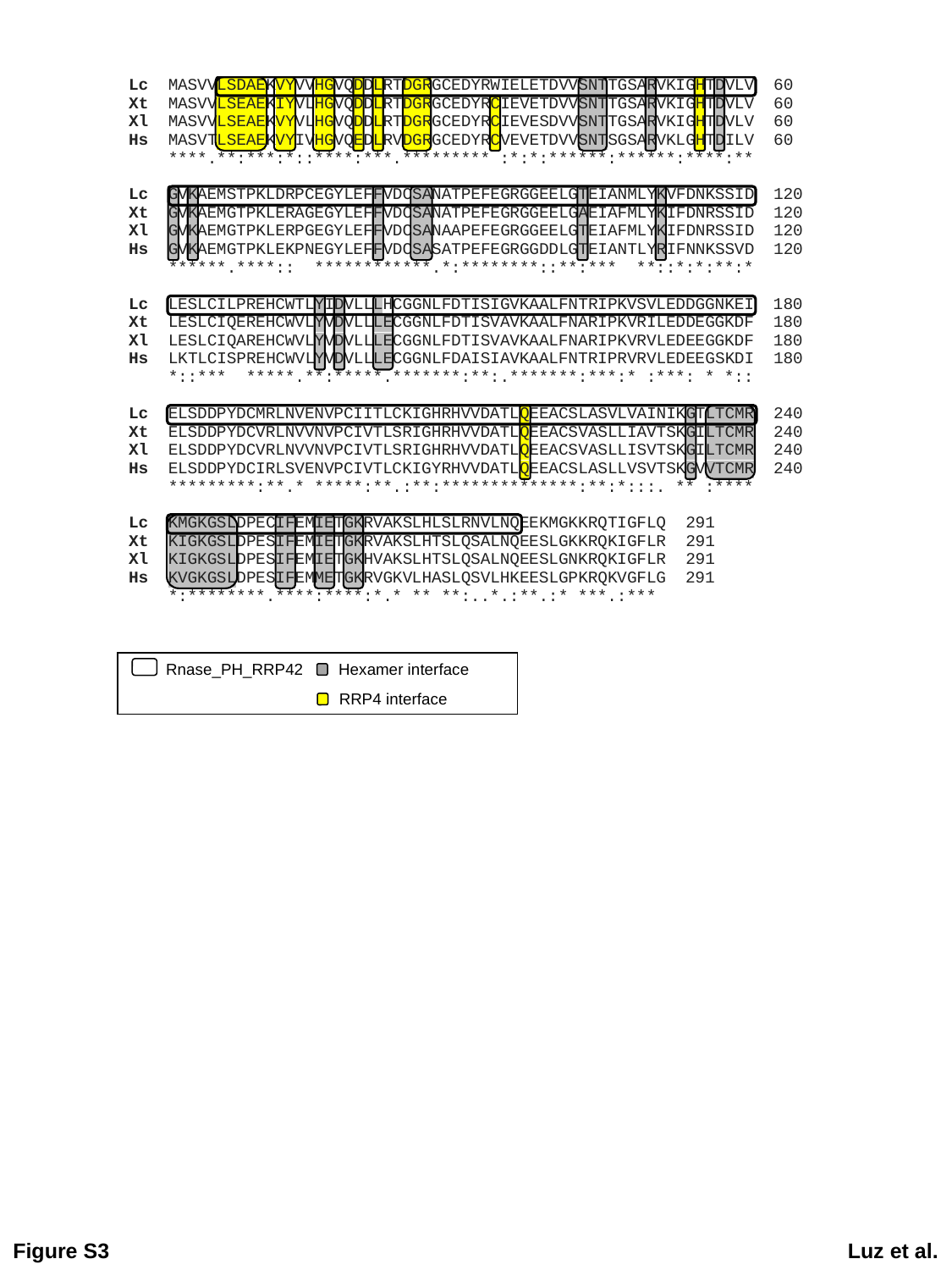

Rnase_PH_RRP42 Hexamer interface
 RRP4 interface
Figure S3
Luz et al.

## Slide 6
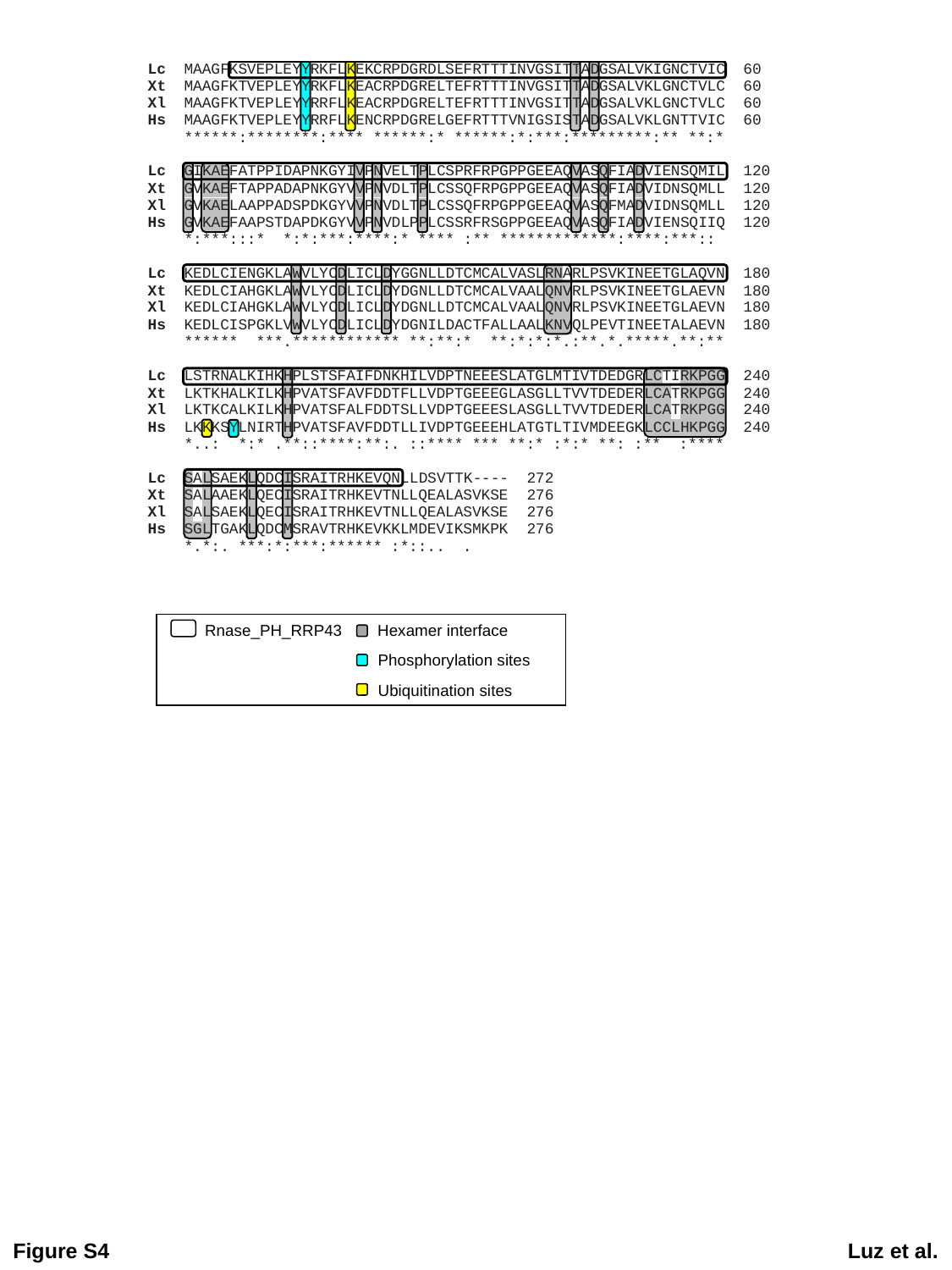

Rnase_PH_RRP43 Hexamer interface
 Phosphorylation sites
 Ubiquitination sites
Figure S4
Luz et al.

## Slide 7
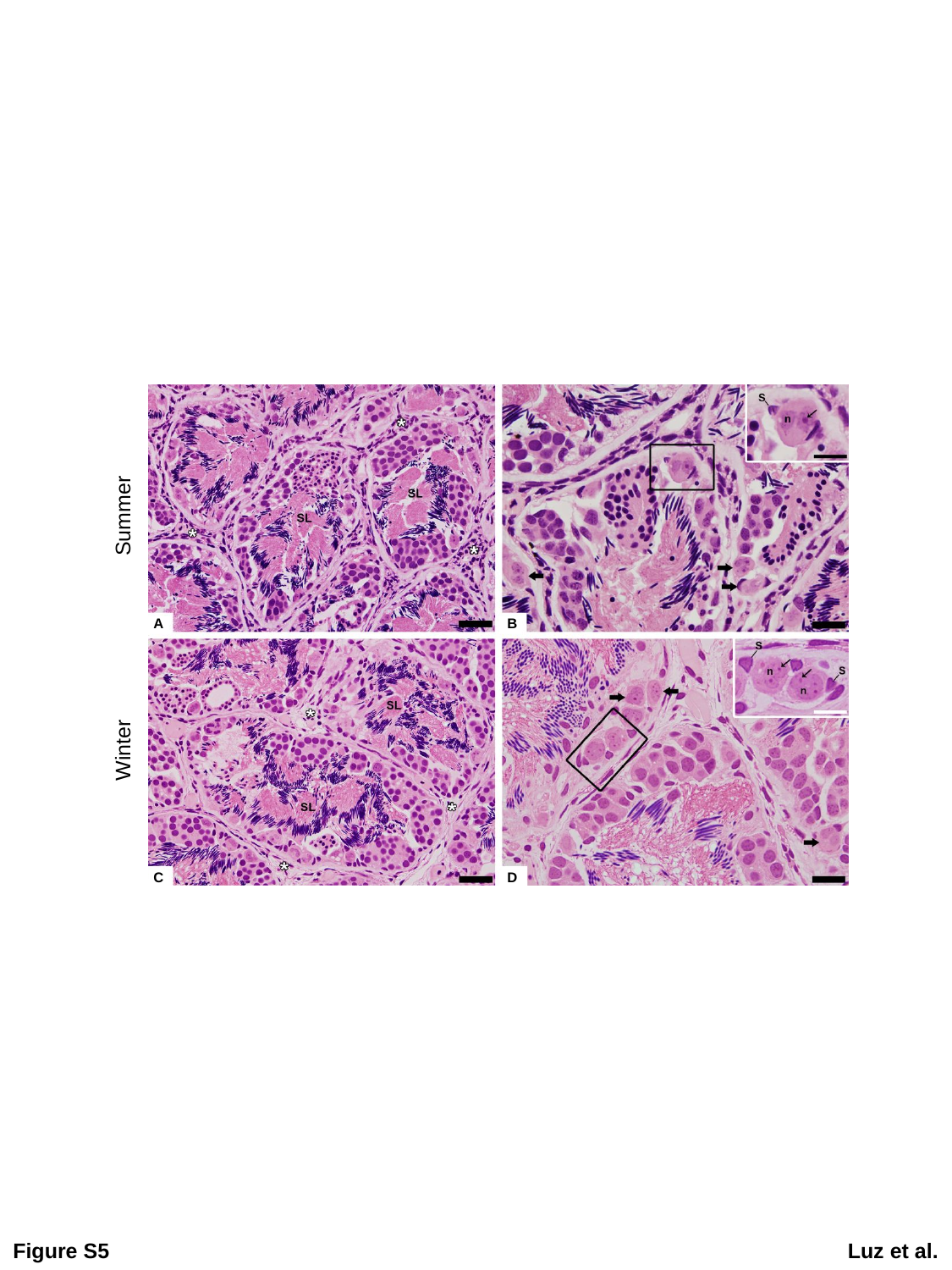

A
B
C
D
Summer
Winter
Figure S5
Luz et al.
